# No turning point? Suicide trends in Brazil and the impact of the COVID-19 pandemic

**DOI:** 10.1017/S2045796026100675

**Published:** 2026-05-04

**Authors:** Lucas Silva, Jorge Artur Peçanha de Miranda Coelho, Valfrido Leão de-Melo-Neto

**Affiliations:** Postgraduate Program in Medical Sciences, Federal University of Alagoas, Maceió, AL, Brazil

**Keywords:** epidemiology, psychometrics, social and political issues, statistics, suicide

## Abstract

**Aims:**

The COVID-19 pandemic has exerted significant mental health impacts worldwide, with a major concern in the literature being its potential effect on suicide rates. Brazil, one of the countries most severely affected by the pandemic, still lacks clear evidence regarding the consequences of the crisis on self-inflicted deaths. This paper aims to estimate the impact of the COVID-19 pandemic on suicide rates in Brazil.

**Methods:**

We employed an interrupted time series design with seasonal adjustments to estimate changes in suicide rates per 100,000 population. The analysis was based on deaths from all forms of self-inflicted injury, as classified by the International Classification of Diseases. We estimated trends for the total population, stratified by sex and administrative region.

**Results:**

Suicide rates increased significantly before the pandemic (*β*₁ = 0.00148, *p* < 0.001). No significant change in trend was observed after the onset of the pandemic at the national level (*β*₃ = 0.00092, *p* > 0.05). Among men, both the pre-pandemic trend (*β*₁ = 0.00236, *p* < 0.001) and the post-pandemic increase (*β*₃ = 0.00155, *p* < 0.05) were significant. For women, the pre-pandemic trend was modest (*β*₁ = 0.00065, *p* < 0.001), and the post-pandemic slope was not significant (*β*₃ = 0.00033, *p* = 0.10). Regionally, the Central-West (*β*₃ = 0.00217, *p* < 0.01) and North (*β*₃ = 0.00186, *p* < 0.05) experienced significant post-pandemic increases, while the Southeast (*β*₃ = 0.00087, *p* > 0.05) and South (*β*₃ = −0.00034, *p* > 0.05) showed no significant changes. Seasonal effects revealed consistent mid-year declines across all groups and regions.

**Conclusions:**

The COVID-19 pandemic did not produce a statistically significant shift in national suicide trends but coincided with the persistence of pre-existing upward patterns in specific demographic and regional contexts. These findings underscore the need for targeted and region-specific suicide prevention strategies.

## Introduction

The COVID-19 pandemic triggered an unprecedented global health and social crisis, with profound consequences for mental health. As fear, grief, social isolation and economic hardship intensified, so did concerns about suicide (Czeisler, 2020; Sher, 2020). Although early studies suggested no immediate rise in suicide rates in high-income countries (Pirkis *et al.*, 2021, 2022), the picture remains unclear in middle-income settings, where public health systems were already under strain.

In Brazil, suicide is a growing concern. Despite a national rate (6.4 per 100,000) below the global average (World Health Organization, 2021), suicides have steadily increased over the past decade – particularly among men and in socially vulnerable regions (Machado and Santos, 2015; Rodrigues *et al.*, 2019). The pandemic unfolded in this already adverse context, marked by political instability (Lancet, 2020), limited coordination in crisis management (Abrucio *et al.*, 2020), and chronic underfunding of mental health services (Trapé and Campos, 2017).

From a theoretical standpoint, several pathways link the pandemic to suicide. Stress-vulnerability models suggest that individuals under chronic stress may be more susceptible to acute events such as unemployment or bereavement (Demke, 2022). Strain theory adds that conflicting social expectations and unmet goals can contribute to suicidal ideation, especially under conditions of economic or interpersonal disintegration (Jie, 2019).

Yet, empirical evidence from Latin America remains scarce. While Brazil has been heavily impacted by COVID-19, few studies have evaluated whether and how suicide rates shifted during the pandemic, and even fewer have examined disaggregated patterns by sex and region.

This study addresses that gap by applying an interrupted time series design to monthly suicide data from 2013 to 2023. We estimate changes in level and trend associated with the pandemic, exploring differential patterns across gender and Brazil’s five major regions.

## Methods

### Data

All deaths due to intentionally self-inflicted injuries, categorized as X60-X84 according to the International Classification of Diseases, were considered for analysis. The period of coverage ranges from 2013 to 2023.

Suicide attempts and any other suicides occurring outside the analysed period are excluded from the analysis. Data for this propose were obtained from the Mortality Information System – SIM (DATASUS, 2024a).

Next, the monthly suicide rate is calculated, which is the ratio of the total number of suicide deaths divided by the population of a given year, multiplied by a factor of 100,000, as illustrated in Equation 1:
(1)
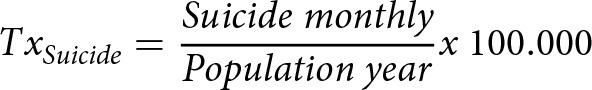


The calculation of the rate is a way to mitigate the population distortions produced by absolute values. Population data were obtained from the Population Projection of the Federative Units by sex, single age or age group: 2010–2060 (2018 edition), conducted by the Brazilian Institute of Geography and Statistics (IBGE, in Portuguese) and made available by the Department of Information and Informatics of the Unified Health System (DATASUS, 2024b).

### Statistical analysis

The interrupted time series design is widely used in evaluating the effectiveness of health interventions implemented at a defined point in time at the population level (Bernal *et al.*, 2017). This method enables establishing an underlying trend through a time series of the outcome of interest, interrupted by an intervention. The hypothetical counterfactual scenario in which the intervention did not occur and the trend continued unchanged is used as a comparison to evaluate the impact of the intervention, analysing any changes that occurred in the post-intervention period (Linden, 2015). Due to this strategy, interrupted time series are considered quasi-experimental methods (Shadish *et al.*, 2002), providing a rigorous and valid comparative analysis to evaluate the effectiveness of health interventions (Wagner *et al.*, 2002).

These techniques possess a great deal of flexibility, allowing for the examination of continuous and discrete outcomes that are measured in regularly spaced intervals (Wagner *et al.*, 2002). Linear regression models are frequently employed in the examination of interrupted time series, offering a robust and easily accessible analytical method for assessing the effectiveness of health interventions (Bernal *et al.*, 2017). Moreover, there exist various expansions of linear regression models that can be employed to enhance the analysis, such as the incorporation of seasonality, autocorrelation and changes in trend over time (Shumway and Stoffer, 2010). In conclusion, interrupted time series analysis represents a powerful and highly adaptable technique for evaluating the impact of health interventions at the population level. It provides a valid and rigorous approach to decision-making in the field of public health.

In the present case, the aim is to analyse the curve of the suicide rate in the country and estimate an underlying trend after the emergence of the pandemic in 2020, which is considered as the intervention of the study. Then, it will be possible to observe if such an event was capable of altering the trend of suicides in the country before its emergence.

From a statistical standpoint, the model is defined as follows:
(2)
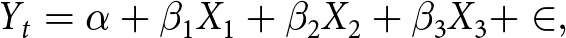
where *Y_t_* represents the annual suicide rate, *X*_1_ is a discrete variable indicating the pre-pandemic trend, *X*_2_ is a fictitious variable representing the immediate level change after the pandemic (0 = before the pandemic, 1 = after the pandemic), and *X*_3_ indicates the change in slope after the pandemic, which is calculated as a multiplicative term between *X*_1_ (time) and *X*_2_ (level). The coefficients are used to measure the effect of the intervention at a specific point in time: *β*_1_ (trend) represents the rate of change over time before the intervention, *β*_2_ (level) indicates the magnitude of the immediate change caused by the intervention, and *β*_3_ (change) measures the difference in the rate of change over time after the intervention.

To assess autocorrelation in model residuals, we applied Durbin–Watson, Breusch–Godfrey, and Ljung–Box tests. Final models were estimated using Newey–West standard errors to correct for autocorrelation and heteroscedasticity. Monthly dummy variables were introduced into the regression model to control for seasonal patterns in suicide rates.

### Subgroup analysis

To elucidate disparities among diverse demographic cohorts, the analyses were categorized according to (a) the general population; (b) gender, and (c) Brazil’s administrative regions (North, Northeast, Southeast, South, and Central-West), thereby allowing for assessment of effect consistency across sexes and identification of regional variations.

### Computational tools

The data was analysed using R Statistical 4.0.5, and all significance tests were two-tailed Replication materials, including raw data and computational scripts, are available at: https://osf.io/chysz/.

## Results

### General population

**Figure 1.**
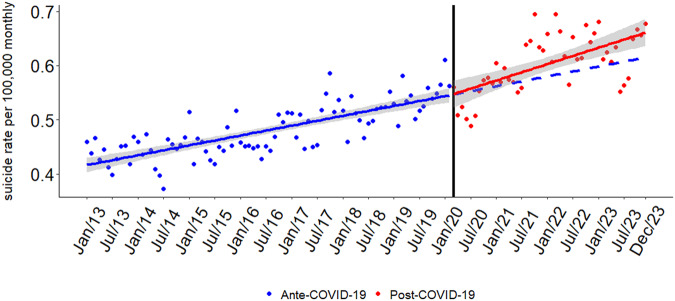
Interrupted time series analysis of monthly suicide rates in Brazil from 2013 to 2023.

Figure 1 displays the monthly suicide rates in Brazil from 2013 to 2023, while Table 1 presents the adjusted regression coefficients with Newey–West robust standard errors. The rate exhibited a steady upward trend prior to the onset of the COVID-19 pandemic (*β*₁ = 0.00148, *p* < 0.001). There was no statistically significant level shift immediately after the pandemic began (*β*₂ = 0.00413, *p* > 0.05), and the post-pandemic trend remained positive but not statistically significant (*β*₃ = 0.00092, *p* > 0.05). These findings suggest a continuation of long-term trends rather than a substantial disruption due to the pandemic. Seasonal patterns were statistically significant, with lower suicide rates consistently observed between April and August. These results suggest that the pandemic did not interrupt the overall upward trajectory in suicide rates.

Caption: The black vertical line marks the intervention point, corresponding to the onset of the COVID-19 pandemic (March 2020). The circular points represent monthly suicide rate observations: blue dots for the pre-pandemic period and red dots for the post-pandemic period. The solid blue line depicts the estimated trend before the intervention, while the solid red line represents the observed trend after the pandemic began. The dashed blue line shows the counterfactual trend – i.e., the expected trajectory had the pandemic not occurred. The grey shaded area corresponds to the 95% confidence interval around the model estimates.

**Table 1.** Estimated coefficients and trends from interrupted time series analysis with Newey–West robust standard errors for the general population

(Intercept)	0.44843***
	(0.00858)
Time	0.00148***
	(0.00012)
Level	0.00413 (0.0165)
Trend	0.00092 (0.00056)
February	−0.04667***
	(0.00765)
March	−0.00719 (0.01089)
April	−0.04023***
	(0.01054)
May	−0.04115***
	(0.00888)
June	−0.07763***
	(0.00912)
July	−0.06899***
	(0.01153)
August	−0.04434***
	(0.01198)
September	−0.02352*
	(0.00983)
October	−0.00311
	(0.01212)
November	−0.02643***
	(0.00711)
December	−0.00685 (0.00945)
Number of observations	132
*R* squared	0.89840
Adj. *R* squared	0.88624

*Notes:* Standard errors in parentheses.

Significance levels: ***p < 0.001; **p < 0.01; *p < 0.05. January is the reference month.

### Sex

**Figure 2.**
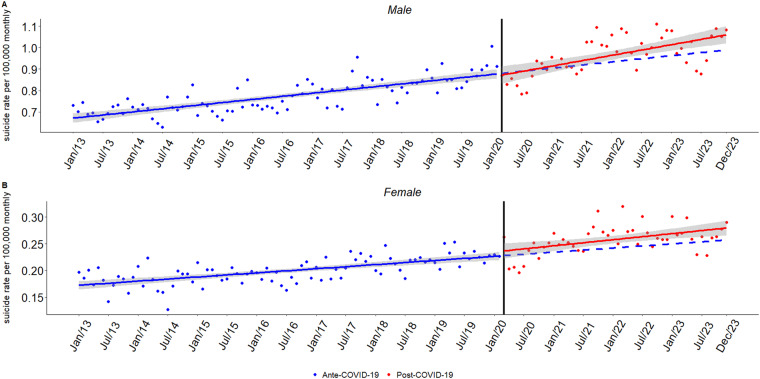
Interrupted time series analysis of monthly suicide rates by sex in Brazil from 2013 to 2023.

*Note*: The black vertical line marks the intervention point, corresponding to the onset of the COVID-19 pandemic (March 2020). The circular points represent monthly suicide rate observations: blue dots for the pre-pandemic period and red dots for the post-pandemic period. The solid blue line depicts the estimated trend before the intervention, while the solid red line represents the observed trend after the pandemic began. The dashed blue line shows the counterfactual trend – i.e., the expected trajectory had the pandemic not occurred. The grey shaded area corresponds to the 95% confidence interval around the model estimates.

Figure 2 presents suicide trends stratified by sex, with corresponding model estimates detailed in Table 2. Among men, suicide rates increased steadily prior to the pandemic (*β*₁ = 0.00236, *p* < 0.001) and continued to rise at a slower, yet statistically significant, pace after March 2020 (*β*₃ = 0.00155, *p* < 0.05). For women, both the pre-pandemic (*β*₁ = 0.00065, *p* < 0.001) and post-pandemic (*β*₃ = 0.00033, *p* > 0.05) trends were milder, with no significant change observed. In both groups, seasonal declines were evident, particularly during the middle months of the year.

**Table 2.** Estimated coefficients and trends from interrupted time series analysis with Newey–West robust standard errors, by sex

	Male	Female
(Intercept)	0.72338***	0.18443***
	(0.01355)	(0.00582)
Time	0.00236***	0.00065***
	(0.00019)	(0.00008)
Level	0.00057	0.0076
	(0.02515)	(0.00964)
Trend	0.00155***	0.00033
	(0.00085)	(0.00033)
February	−0.07207***	−0.0223***
	(0.0136)	(0.00437)
March	−0.02659	0.01136
	(0.01758)	(0.00704)
April	−0.06878***	−0.01269*
	(0.01875)	(0.00595)
May	−0.07006***	−0.01327*
	(0.01402)	(0.00714)
June	−0.12432***	−0.03282***
	(0.01511)	(0.006)
July	−0.1103***	−0.02944***
	(0.01949)	(0.00856)
August	−0.07516***	−0.01473*
	(0.0207)	(0.00704)
September	−0.03723*	−0.01031
	(0.01675)	(0.00632)
October	0.00281	−0.0086
	(0.02084)	(0.00759)
November	−0.04021**	−0.01317*
	(0.01203)	(0.00578)
December	−0.00231	−0.01114*
	(0.01712)	(0.00566)
Number of observations	132	132
*R* squared	0.89083	0.80473
Adj. *R* squared	0.87777	0.78137

*Notes:* Standard errors in parentheses.

Significance levels: ***p < 0.001; **p < 0.01; *p < 0.05. January is the reference month.

### Regions

**Figure 3.**
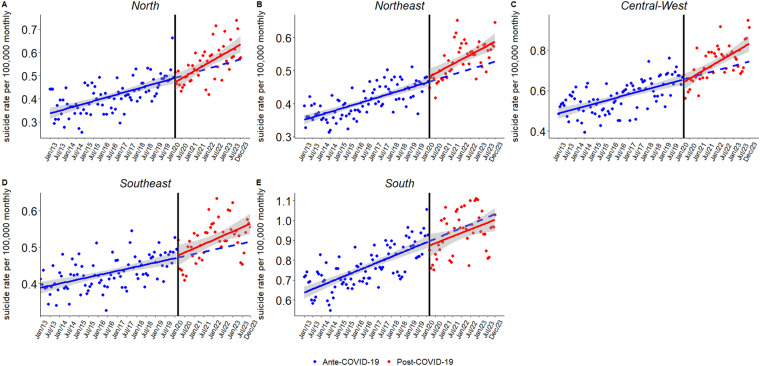
Interrupted time series analysis of monthly suicide rates by region in Brazil from 2013 to 2023.

*Note*: The black vertical line marks the intervention point, corresponding to the onset of the COVID-19 pandemic (March 2020). The circular points represent monthly suicide rate observations: blue dots for the pre-pandemic period and red dots for the post-pandemic period. The solid blue line depicts the estimated trend before the intervention, while the solid red line represents the observed trend after the pandemic began. The dashed blue line shows the counterfactual trend – i.e., the expected trajectory had the pandemic not occurred. The grey shaded area corresponds to the 95% confidence interval around the model estimates.

Figure 3 illustrates regional variations, with model coefficients reported in Table 3. The Central-West region exhibited the steepest post-pandemic increase (*β*₃ = 0.00217, *p* < 0.01), followed by the North (*β*₃ = 0.00186, *p* < 0.01) and the Northeast (*β*₃ = 0.00099, *p* < 0.05). These trends reflect sustained upward trajectories in suicide rates, with no significant level shifts. In contrast, the Southeast (*β*₃ = 0.00087, *p* > 0.05) and South (*β*₃ = −0.00034, *p* > 0.05) showed non-significant post-pandemic trends. Seasonal variation remained a consistent feature across all regions.

**Table 3.** Estimated coefficients and trends from interrupted time series analysis with Newey–West robust standard errors, by regions

	North	Northeast	Southeast	South	Central-West
(Intercept)	0.35384***	0.36803***	0.42115***	0.72245***	0.48483***
	(0.02658)	(0.01158)	(0.00987)	(0.01576)	(0.02135)
Time	0.00179***	0.00134***	0.00093***	0.00294***	0.00193***
	(0.0003)	(0.00014)	(0.00012)	(0.00027)	(0.00028)
Level	−0.02109	0.01491	0.01002	−0.00843	−0.01282
	(0.01664)	(0.01796)	(0.01638)	(0.0324)	(0.02143)
Trend	0.00186**	0.00099*	0.00087	−0.00034	0.00217**
	(0.00056)	(0.00052)	(0.0006)	(0.00119)	(0.00081)
February	−0.0487*	−0.04181***	−0.04428***	−0.06774***	−0.03621
	(0.02701)	(0.01054)	(0.01059)	(0.01992)	(0.02525)
March	0.00016	−0.01279	0.00109	−0.04515*	0.02899
	(0.0257)	(0.01401)	(0.01375)	(0.02037)	(0.02107)
April	−0.04256	−0.01885*	−0.04595***	−0.09522***	0.01928
	(0.02786)	(0.00969)	(0.01323)	(0.02464)	(0.02307)
May	0.00158	−0.00927	−0.04928***	−0.13426***	0.01258
	(0.02914)	(0.01091)	(0.00972)	(0.01971)	(0.02366)
June	−0.02535	−0.03426*	−0.08747***	−0.18167***	−0.04428*
	(0.02817)	(0.01335)	(0.01131)	(0.01836)	(0.02635)
July	−0.02273	−0.04204**	−0.07026***	−0.15068***	−0.05901**
	(0.03003)	(0.01463)	(0.01355)	(0.02669)	(0.02181)
August	−0.01537	−0.00457	−0.04978***	−0.13805***	−0.01479
	(0.02911)	(0.01448)	(0.01254)	(0.02139)	(0.02373)
September	−0.02366	−0.01789	−0.01403	−0.08504***	0.01812
	(0.02946)	(0.01231)	(0.0132)	(0.02171)	(0.02205)
October	−0.00293	0.0076	0.00736	−0.08497**	0.05275**
	(0.02905)	(0.01484)	(0.01355)	(0.02711)	(0.01713)
November	−0.03864	−0.03619**	−0.02443*	−0.03411*	0.02485
	(0.03747)	(0.0116)	(0.01136)	(0.01851)	(0.02096)
December	−0.0047	−0.00342	−0.01211	−0.0184	0.02861
	(0.03143)	(0.0103)	(0.01471)	(0.02524)	(0.02676)
Number of observations	132	132	132	132	132
*R* squared	0.70360	0.82845	0.78969	0.79185	0.77653
Adj. *R* squared	0.66813	0.80792	0.76453	0.76694	0.74979

*Notes:* Standard errors in parentheses.

Significance levels: ***p < 0.001; **p < 0.01; *p < 0.05. January is the reference month.

## Discussion

This study assessed the effect of the COVID-19 pandemic on suicide rates in Brazil using interrupted time series analysis. Our results show a persistent upward trend in suicide rates before the pandemic, with no immediate level shift after March 2020 but a continued increase, particularly in certain groups and regions. These findings align with previous national studies reporting rising suicide mortality in Brazil over the past two decades (Cruz *et al.*, 2023; Silva *et al.*, 2025), attributed to chronic underinvestment in mental health, inequities in service access, and persistent social stigma (Fukuda *et al.*, 2016; Bezerra and Barbosa, 2023).

Although national efforts such as Brazil’s Yellow September campaign (Associação Brasileira de Psiquiatria, 2024) and mandatory suicide reporting (Brasil, 2019) sought to improve prevention and surveillance, their impact remains unclear. Greater professional awareness may have improved reporting accuracy, but implementation gaps – such as insufficient training, poor intersectoral coordination, and resource constraints – likely limited the effectiveness of these initiatives (Pereira *et al.*, 2024).

The continuation of the pre-existing upward trend after the onset of the pandemic supports global evidence suggesting that COVID-19 did not immediately raise suicide rates (Pirkis *et al.*, 2021, 2022). However, the gradual post-pandemic increase observed in our study suggests a delayed impact, possibly due to prolonged social isolation, disruptions in mental health care, and worsening socioeconomic conditions. This trajectory mirrors patterns identified in countries such as Japan, India, and the US, where suicide rates rose after an initial phase of stability (Wasserman, 2022).

Gender differences were pronounced. Among men, the upward trend persisted post-pandemic, while women showed a milder and statistically non-significant increase. This echoes prior evidence that economic stressors, job instability, and lower help-seeking behaviour disproportionately affect men (Borges *et al.*, 2023). In contrast, women may benefit from stronger social support networks and greater mental health service use, though this varies cross-nationally (Pirkis *et al.*, 2022). These patterns underscore the importance of gender-responsive suicide prevention strategies.

Regional disparities also emerged. The Central-West, North, and Northeast regions showed the steepest post-pandemic increases in suicide rates, while trends remained stable in the South and Southeast. These findings highlight the unequal burden of suicide across Brazil and suggest that structural vulnerabilities may have amplified the effects of the pandemic in underserved regions (Ornell *et al.*, 2022; Trettel *et al.*, 2022). These outcomes may also have broader relevance for other low- and middle-income countries (LMICs) facing similar structural challenges. In many such contexts, suicide patterns are shaped not only by acute crises but also by persistent socioeconomic inequalities, uneven distribution of mental health services, and barriers to access in rural or underserved areas (Iemmi *et al.*, 2016). In this sense, the persistence of pre-existing trends observed in our analysis may reflect structural determinants that extend beyond the Brazilian case. Targeted mental health policies are urgently needed to address these regional disparities.

Seasonal patterns were consistent throughout the study period. Suicide rates declined during mid-year months across all groups and regions. These findings corroborate prior research linking suicide to climatic factors, including sunlight exposure and temperature variation, which may influence biological rhythms and mood regulation (Gao *et al.*, 2019; Freichel and O’Shea, 2023).

The absence of a clear structural break in national suicide trends following the onset of the pandemic deserves careful interpretation. Rather than producing an immediate turning point, the pandemic appears to have coincided with the continuation of pre-existing trajectories. One possible explanation is that short-term social cohesion and collective coping responses may have temporarily mitigated suicide risk during the early months of the pandemic. Alternatively, the persistence of long-standing structural determinants may have exerted a stronger influence on suicide trends than the acute shock associated with COVID-19. These findings suggest that the pandemic should be interpreted not as an isolated driver of suicide mortality, but as an event interacting with pre-existing structural vulnerabilities.

From a policy perspective, the persistence of increasing suicide trends underscores the importance of strengthening prevention strategies that address both clinical and structural determinants of suicide risk. Expanding access to community-based mental health services remains a critical priority. Integrating systematic screening for depression and suicidal ideation within primary health care could facilitate earlier identification of individuals at risk, while strengthening crisis intervention services and surveillance systems may improve the capacity to respond to emerging patterns of suicide mortality. In addition, broader social policies aimed at reducing economic insecurity and improving access to social protection programmes may also play a role in mitigating suicide risk in vulnerable populations.

Despite its strengths, this study has limitations. First, suicide data may be affected by underreporting and misclassification. Second, although we addressed autocorrelation and seasonality, the ITS design cannot fully isolate all time-varying confounders. Nonetheless, the use of stratified models and robust standard errors strengthens the reliability of our inferences.

Overall, the pandemic did not reverse pre-existing trends but may have compounded structural risk factors already in place. Our findings reinforce the urgency of scaling up suicide prevention policies in Brazil, with special attention to regional and demographic inequities.

## Conclusion

This study identified persistent upward trends in suicide rates during the COVID-19 pandemic in Brazil, with marked regional and gender-specific variations. These findings reinforce the need for sustained surveillance and targeted mental health interventions, particularly in regions and demographic groups where vulnerabilities have intensified. As the long-term mental health consequences of the pandemic continue to emerge, suicide prevention efforts must prioritize evidence-based, context-specific strategies that are sensitive to local disparities in access, stigma and care delivery. Addressing these challenges is essential to reducing suicide risk and promoting mental health equity across the country.

## Data Availability

Replication materials, including raw data and computational scripts, are available at: https://osf.io/chysz/.
